# The effect of sexual health counseling on women's sexual satisfaction in postpartum period: A randomized clinical trial

**DOI:** 10.18502/ijrm.v17i1.3819

**Published:** 2019-03-07

**Authors:** Maryam Zamani, Robab Latifnejad Roudsari, Maryam Moradi, Habibollah Esmaily

**Affiliations:** ^1^Department of Midwifery, School of Nursing and Midwifery, Mashhad University of Medical Sciences, Mashhad, Iran.; ^2^Department of Midwifery, School of Nursing and Midwifery, Neyshabur University of Medical Sciences, Neyshabur, Iran.; ^3^Research Centre for Patient Safety, Mashhad University of Medical Sciences, Mashhad, Iran.; ^4^Nursing and Midwifery Care Research Centre, Mashhad University of Medical Sciences, Mashhad, Iran.; ^5^Social Determinants of Health Research Center, Mashhad University of Medical Sciences, Mashhad, Iran.

**Keywords:** *Sexual satisfaction*, * Sexual counseling*, * Sexual health*, * Postpartum.*

## Abstract

**Background:**

Many couples experience decreasing sexual satisfaction in postpartum period. Various sexual health counseling approaches have been designed for postpartum women to address their common sexual concerns and problems.

**Objective:**

This study aimed to investigate the effectiveness of Women's Postpartum Sexual Health Program (WPSHP) on women's sexual satisfaction in postpartum period.

**Materials and Methods:**

The study was a single blind randomized clinical trial on 75 postpartum women aged 18–35 yr with low sexual satisfaction who attended urban health-care centres in Mashhad, Iran in 2016. Data were collected using a demographic questionnaire, the Depression Anxiety Stress Scales-21, and the Larson Sexual Satisfaction Questionnaire. The intervention group received counselling based on the WPSHP, a four-session, group- and couples-based program. The control group just received postpartum routine care.

**Results:**

Both the intervention and control groups were homogeneous for demographic variables. According to the Mann-Whitney test, sexual satisfaction score in the intervention group was significantly higher than the control group 8 weeks after the intervention (p < 0.001). According to the Wilcoxon test, there was a significant difference in the mean score of sexual satisfaction before and after intervention in the intervention group (p < 0.001).

**Conclusion:**

WPSHP caused higher levels of sexual satisfaction. It is therefore recommended to use this program in women during the postpartum period to promote their sexual satisfaction.

## 1. Introduction

Sexual satisfaction, as a subjective assessment tool, involves effective reactions which are, generally, resulted from sexual relationships and influence on one's general health from numerous aspects (1, 2). Women in the postpartum period face many changes such as sleep deprivation, postpartum hormonal changes, lactation, as well as the recovery of health and the potentially painful and prolonged effects of vaginal delivery or complications of cesarean sectional. These factors can affect their physical, emotional, and sexual health (3). Many couples report reduced sexual satisfaction of relationship which is mostly related to their impaired sexual relationships (4). Several studies have shown a decrease in sexual satisfaction in the postpartum period (5–7). Sexual problems have a great adverse effect on the different life aspects of women; among them are their self-image, self-confidence, sense of health, and satisfaction of relationship (8, 9).

Women have high desire to be supported and receive more information about postnatal sexual issues from their care providers (10–15). Lack of sexual health counseling after childbirth is one of the important factors leading to disordered sexual function during postnatal period (16). Although sexual instruction education after childbirth is important, but currently its content is usually focused on the time of the first intercourse and choosing postpartum contraceptive methods, which does not meet the women's needs in their whole postpartum period (17).

There are insufficient number of sexual counseling approaches for women at their postpartum period such as Permission, Limited Information, Specific Suggestion, Intensive Treatment (PLISSIT) and Bring up, Explain, Tell, Time, Educate, Record (BETTER), which have been recommended to be used in this period (17, 18). However, these approaches are more focused on physical or biological aspects of sexuality and are not designed specifically for postnatal women. So, there is a need for sexual counseling methods that consider relationships and psychosocial elements of the women's life (8, 19–21). Postpartum period creates unique challenges in interpersonal and sexual relationships and causes bio-psychological and social changes in one's life (22). As a result, counseling in this course requires a multidisciplinary approach.

The Women's Postpartum Sexual Health Program (WPSHP), which has been recommended by McBride and his colleagues (2017), considers all these aspects of women's life. This program is designed with a multidisciplinary approach to women's sexual counseling in the postpartum period, but its effectiveness on sexual satisfaction has not been investigated in research so far (23). Sexual counseling is one of the eight elements of the family health and is one of the midwifery responsibilities (24). The WPSHP is possible to be delivered by midwives (23).

This study aimed to investigate the effectiveness of sexual health counseling based on WPSHP on woman's sexual satisfaction in postpartum period.

## 2. Materials and Methods

This randomized single-blind clinical trial was conducted during 2016 on 80 postnatal women who attended health-care centers three months to one year after childbirth in Mashhad, Iran in 2016. Multistage cluster random sampling method was used to select the subjects from January 2016 to the end of July 2016. For this purpose, five large health centers in Mashhad were considered as the cluster, from which two large centers were selected using simple random sampling method. Then two urban health centers were randomly selected from the list of the centers covered by each large center (four centers in total), from which two were allocated to the control and two to the intervention groups. Then, in each center the eligible subjects were conveniently selected and allocated to the intervention and control groups.

The study included Iranian women who were the residents of Mashhad, aged between 18 and 35 years, had at least a high school diploma, were married and the only spouse of their husband, living with their spouses together, and recommenced their sexual intercourse after delivery. They also had a healthy, singleton, and term newborn in recent childbirth. Women had a low sexual satisfaction (a score of 51–75 based on the Larson Sexual Satisfaction Questionnaire, and their score of stress, anxiety, and depression were less than 17, 9, and 13, respectively, according to the Depression Anxiety Stress Scales-21. Other inclusion criteria included no history of drug addiction or drinking alcohol and no diagnosed mental and psychological problems such as severe depression, delirium, severe anxiety, and obsessive-compulsive disorder. Also women did not suffer from late postpartum hemorrhage, postpartum infection, thromboembolic disorder, pelvic detachment, and mastitis. Finally, women included in the study should not take prescriptions like thiazide diuretics, antihypertensives, anti-depressant lithium, antipsychotics, antihistamines, barbiturates, benzodiazepines, hallucinations, amphetamines, cocaine, anticonvulsants, cimetidine, danazol, digoxin, and levodopa, which affect sexual function, and should not have medical and pelvic surgery like colporrhaphy or reconstructive surgery and also radiation on the reproductive system.

The exclusion criteria included becoming pregnant, breaking sexual intercourse, receiving comprehensive sex education, confronting with a stressful event during the past six months (parent's death, child's death, expulsion from school or work, bankruptcy, and broken marriage) during the study, and attending less than three counseling sessions.

**Figure 1:**
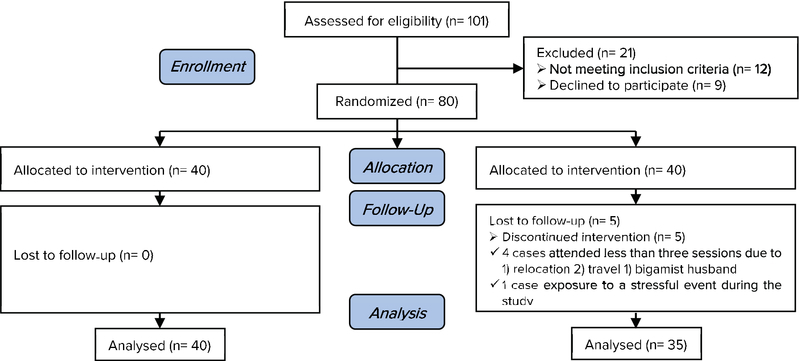
The Consort 2010 Flow Diagram.

To calculate the sample size, the formula for comparing two independent means was employed by taking a confidence interval of 95% and test power of 80%. The minimum sample size was estimated as 32. Considering attrition, the sample size was considered to be 40 in each group. A total of 75 patients completed the study: 35 in the intervention group and 40 in the control group (Figure 1).

Data collection tools in this study included a demographic questionnaire, the Depression Anxiety Stress Scales-21, and the Larson Sexual Satisfaction Questionnaire. Demographic questionnaire included five parts of personal, marital, pregnancy-related, postpartum, and sex-related data. The content validity of demographic questionnaire was verified by some experts in Mashhad University of Medical Sciences in the fields of midwifery, reproductive health, and psychology. Larson's sexual satisfaction questionnaire involves 25 items with answers in 5-point Likert scale (1=not at all, 2=seldom, 3=sometimes, 4=often, 5=always). Total score of sexual satisfaction was between 25 and 125 with scores of <50 represent for sexual dissatisfaction, 51–75 for low satisfaction, 76–100 for moderate satisfaction, and >101 for high satisfaction. Also, its reliability was verified with r=0.82. The validity of sexual satisfaction questionnaire was verified by Larson and his colleagues. The reliability of Larson's questionnaire was also determined by Bahrami *et al*. with Cronbach alpha=0.70, which was reported as r=0.93 using inner consistency (25). Also, their reliability was verified with r=0.82 in this study.

The present study also applied a short form of DASS, called DASS-21, which is a self-report 4-point Likert scale graded from zero to three and composed of 21 questions. The score would be zero if the item does not apply to the individual, 1 or 2 when the item applies sometimes or relatively most of the times; and 3 (maximum) if the item applies in most cases to the individuals. The maximum score in each of the subscales is 21. The scores of 0–14 indicate normal, 15–18 mild, and 19–21 moderate and severe stress. Additionally, the scores of 0–7 indicate normal, 8–9 mild, and 10–21 moderate and severe anxiety. Also, the scores of 0–9 indicate normal, 10–13 mild, and 14–21 moderate to severe depression. Asghari *et al*. in Iran in 2008 confirmed the validity and reliability of DASS-21 (26). The reliability of this tool was also determined in this study (r=0.91).

To collect the data, the postpartum women who attended health-care centers for receiving newborn vaccinations or other services were invited to participate in the study. At first, they completed demographic and DASS-21 questionnaires. Then, women who met the inclusion criteria and agreed to participate were invited to a quiet and private room in health-care center and given some information about how to complete the questionnaires. Both the intervention and control groups received center's routine healthcare services. Subjects in intervention group received four sessions of sexual counseling based on WPSHP program, including three sessions of group and one session of couple counseling. The three first sessions were held by the researcher under supervision of project supervisor and a sex therapist, as a group counseling, comprising 6–8 members. Each session lasted 90 minutes. The last session was held with the attendance of wife and husband, as a couple counseling session, and lasted for 60–90 min. During the first session, the women's cycle of sexual responses after delivery was discussed. The second session was devoted to discuss biological, psychological, and social factors influencing sexual issues after delivery, which was held with a week's time lapse. After two weeks of the last session, the third session was held, which was focused on effective communication skills and intimacy. Finally, the fourth session with two- to three-week interval was guided with the aim of personal support and answering questions of wife and her husband (Table I). After the end of eight weeks of intervention, the Larson Sexual Satisfaction Questionnaire was completed by subjects and collected by the researcher who was unaware of how to allocate the subjects to the groups. Women with severe and moderate depression were referred to the psychiatrist for treatment. After the completion of the study, the counseling was also conducted in the control group, if they desired.

### Ethical considerations

This research is a part of a larger clinical trial approved by the Ethics Committee of Mashhad University of Medical Sciences, Mashhad, Iran. It was registered in the Iranian Registry of Clinical Trials (IRCTID: IRCT2016062228575N). All the participants signed informed consent form and were assured regarding anonymity and confidentiality of their responses. They could also withdraw from the study without any prejudice to their future care.

### Statistical analysis

Data analysis was done using SPSS (Statistical Package for the Social Sciences, version 16.0, SPSS Inc, Chicago, Illinois, USA) using Kolmogorov-Smirnov, Mann-Whitney U test, independent *T*-test, Paired *T*-test, Chi-Square, and Fisher's exact tests. The significance level considered was p < 0.05.

## 3. Results

The subjects in both the groups had no significant difference in terms of demographic variables such as age, education, husband's age, husband's education, job, intention for pregnancy, and duration of marriage. They were also not different in terms of the birth weight of newborn, the time of first intercourse after delivery, marital adaptation, stress, anxiety, and depression scores, perineal tears or episiotomy, complications of perineal tears, contraceptive methods, infant's feeding methods, daily average frequency of breastfeeding, average frequency of intercourse per week, history of taking any sexual educational program, and the history of sexual function before delivery. They also had no history of substance or alcohol addiction, mental problems after delivery, special medical diseases, intense marital conflicts, traumatic events during the last 6 months, history of pelvic surgery, and using drugs influencing sexual function. Demographic characteristics of the subjects have been shown in Table II.

According to the Kolmogorov-Smirnov test, intervention group had normal (p = 0/091) and control group had non-normal distribution of data (p = 0.023). Results of Mann-Whitney U test showed that sexual satisfaction score was not significantly different between the intervention and the control groups at the beginning of the study (p = 0.395). After the intervention, there was a significant difference between the two groups with respect to the sexual satisfaction score (p = 0.028). The results of Wilcoxon test showed that there is no significant difference in sexual satisfaction score before and after the intervention (p = 0.414). However, in the intervention group, a significant difference was seen before and after the intervention (p < 0.001). Also, there was a significant difference between the control and intervention groups in respect of average changes of sexual satisfaction score, before and after intervention (p < 0.001) (Table III). In terms of sexual satisfaction questions, the highest difference was related to the score of question 19: "I feel that sexual relations will make our emotional connection stronger," whereas, the scores related to the questions 11, 15, and 18 had no changes after intervention. The statistical analysis showed that eight weeks after intervention, the scores of stress, anxiety, and depression in the intervention group was significantly lower than the control group (p = 0.04, p = 0.01, p = 0.008).

**Table 1:** The outline of the Women's Postpartum Sexual Health Program (WPSHP) sessions.


**No.**	**Purpose**	<**The Facilitator and Duration**	**Content**	**Tasks**
**1**	To normalize women's postpartum experiences, To introduce Basson's (2000) model of female sexual response	Conducted by a midwife, 90 minutes	Female sexual response cycle and postpartum period	Introducing the female sexual response model to the partner and discussing the suitability of this model with their relationship
**2**	To provide psych education and strategies to begin addressing common bio psychosocial impediments to women's sexual relationship	Conducted by a midwife, 90 minutes	Biopsychosocial factors affecting sexuality	(1) Completing the personal snowball of underlying beliefs, emotional outcomes, and behaviors associated with current sexual intercourse (2) Identifying and replacing problematic thoughts/cognitive deviations through the worksheet, and (3) Discussing and practicing sensate focus (Phase 1)
**3**	Focusing more specifically on intimacy	Conducted by a midwife, 90 minutes	Intimacy and effective communication	Discussing and practicing sensate focus (Phase 2)
**4**	To provide brief, personalized support to each participating woman and her partner	Conducted by a sex therapist and a midwife, 60–90 minutes	The impact of relational factors on sexual issues and strategies to improve each spouse's sexual relationships	Answering questions, providing additional recommendations and strategies

**Table 2:** Frequency distribution of demographic characteristics in intervention and control groups.


**Variable**	**Intervention group**	**Control group**	**p-value**
Women's age*	29.5 ± 4.3	29.4 ± 4.2	0.90a
Husband's age*	34.2 ± 6.8	34.2 ± 6.8	0.85a
Number of childbirth *	1.4 ± 0.5	1.5 ± 0.5	0.98b
Duration of marriage *	7.9 ± 4.3	9.1 ± 4.5	0.24a
Stress	14.1 ± 4.0	15.0 ± 3.4	0.31a
Anxiety	10.8 ± 2.2	11.1 ± 2.3	0.62b
Depression	10.5 ± 3.4	11.6 ± 3.1	0.08b
Mode of delivery **		0.439d
Vaginal delivery	18 (51.4)	17 (42.5)	
Cesarean section	17 (48.6)	23 (57.5)	
Husband’s support**		0.052b
Very low	3 (8.6)	1 (2.5)	
Low	5 (14.3)	2 (5.0)	
Mild	19 (54.3)	20 (50.0)	
Satisfactory	8 (22.9)	17 (42.5)	
Having separate bedroom**		0.647d
Yes	18 (51.4)	23 (57.5)	
No	17 (48.6)	17 (42.5)	
Types of Contraceptives**		0.739c
Linesterol	3 (8.6)	4 (10.0)	
IUD	2 (5.7)	2 (5.0)	
Condom	14 (40.0)	11 (27.5)	
Withdrawal	6 (17.1)	5 (12.5)	
Vasectomy	3 (8.6)	4 (10.0)	
Without contraception	7 (20.0)	14 (35.0)	
Note: *Data presented as mean ± SD;			
**Data presented as *n* (%);			
a=Independent *T*-test b=Mann-Whitney c=Exact x${}^{2}$ d=Chi-square.			

**Table 3:** Frequency distribution of pre- and post-intervention scores of sexual satisfaction in intervention and control groups.


**Sexual satisfaction score**	**Intervention group**	**Control group**	**Mann–Whitney U Test**
Baseline	71.68 ± 12.97	69.20 ± 11.16	Z= –0.850
		p = 0.395
Eight weeks after intervention	75.60 ± 10.37	69.25 ± 11.17	Z= –2.196
		p = 0.028
Difference between pre and post intervention	3.91 ± 5.27	0.05 ± 0.38	Z= –5.490
		p < 0.001
Wilcoxon test statistics	Z= –4.297	Z= –0.816	
	p < 0.001	p = 0.414	
Note: Data presented as mean ± SD.			

## 4. Discussion

The results of study showed that sexual counseling based on the `WPSHP' after eight weeks of intervention increased, significantly, the spouses' sexual satisfaction in the intervention group compared to the control group. In a clinical randomized trial on 34 spouses to investigate the effect of four sessions of psycho-education (each lasting for 120 min) on the spouses' sexual satisfaction, Karimi and colleagues (2013) found that after three months of intervention, sexual satisfaction of spouses who received sexual health education was higher than the control group, which was consistent with the results of this study. Subjects with any level of sexual satisfaction entered in this study (24). The reason for the same results might be the similarity of intervention applied in both the studies including the number of sessions and the attendance of husband in the counseling sessions. The study by Yörük and colleagues showed a limited impact of the PLISSIT Model intervention on the reduction of sexual problems experienced by women during the postpartum period (18). Hipp and colleagues indicated that health-care providers should discuss the social and relationship aspects of sexuality instead of just addressing the physical issues (27). In the current study, the highest score of questions was seen in relation to question 19: "I feel that sexual relations will make our emotional connection stronger." In this relation, Basson cycle reported that proper women's sexual stimulation increases emotional intimacy (28).

Creating interactive sexual relationships between couples in the postpartum period could improve sexual health and resulted in a higher level of intimacy (9). The positive results obtained in this study can be due to several reasons. Counseling program applied in this study was focused on psychosocial dimensions of sexuality and skills of getting sexual satisfaction based on the improvement of interpersonal relationships. The education of communication skills, including psycho-educational issues about sexual relationships, supportive and motivated behaviors, as well as conflict management skills, helps spouses in their transition to parenting and, finally, increase their sexual satisfaction (23).

In this study, group counselling helped in the normalization of sexual problems and concerns in postpartum period, so that mothers realized that they are not the only ones who suffer from these problems. Another benefit of the program was having a paired couple counseling session that addressed the sexual concerns of spouses as well. Also, this program reduced stress, anxiety, and depression of postpartum women which, in turn, seems to increase their sexual satisfaction (29). Zamani *et al* have also recommended adopting effective counselling approaches to decrease sexual distress in postpartum period (30). In the study by Gamble, a brief midwife-led counseling intervention for women, who reported a distressing birth experience, was effective in reducing the symptoms of trauma, depression, and stress (31).

This research had some limitations. One of those was everyday minor stressors in the family and society and the individual differences to manage them, which could affect the acceptance of the issues discussed within the counseling sessions. Also, a complete control of other influencing factors like media, neighbors, relatives, and friends was not possible. Furthermore, it was quiet hard to monitor exactly the quality of homework exercises done by the subjects, but it was tried to relatively monitor them during the period of intervention using daily checklists. Regarding the future research, it is recommended to compare the impact of `WPSHP' with other sexual counseling approaches such as BETTER and PLISSIT model.

## 5. Conclusion

Offering sexual counselling based on `WPSHP' caused enhanced sexual satisfaction in women who experienced decreased sexual satisfaction in the postpartum period. Therefore, it is recommended to use this program in postpartum women with decreased sexual satisfaction.

##  Conflict of Interest

The authors declare that there is no conflict of interest in relation to the publication of this article.
